# Biological cellulose saccharification using a coculture of *Clostridium thermocellum* and *Thermobrachium celere* strain A9

**DOI:** 10.1007/s00253-022-11818-0

**Published:** 2022-02-14

**Authors:** Sreyneang Nhim, Rattiya Waeonukul, Ayaka Uke, Sirilak Baramee, Khanok Ratanakhanokchai, Chakrit Tachaapaikoon, Patthra Pason, Ya-Jun Liu, Akihiko Kosugi

**Affiliations:** 1 School of Bioresources and Technology, King Mongkut’s University of Technology Thonburi (KMUTT), 10150 Bangkok, Thailand; 2 Excellent Center of Enzyme Technology and Microbial Utilization, Pilot Plant Development and Training Institute (PDTI), King Mongkut’s University of Technology Thonburi (KMUTT), Bangkok, 10150 Thailand; 3 Biological Resources and Post-harvest Division, Japan International Research Center for Agricultural Sciences (JIRCAS), 1-1 Ohwashi, Tsukuba, Ibaraki 305-8686 Japan; 4 CAS Key Laboratory of Biofuels, Qingdao Institute of Bioenergy and Bioprocess Technology, Chinese Academy of Sciences, Qingdao, 266101 People’s Republic of China; 5 Shandong Energy Institute, Qingdao, 266101 People’s Republic of China; 6 Qingdao New Energy Shandong Laboratory, Qingdao, 266101 People’s Republic of China

**Keywords:** Biological saccharification, *Thermobrachium celere*, *Caloramator celer*, *Clostridium thermocellum*, β-Glucosidase, Glucose tolerance, Thermostability

## Abstract

**Abstract:**

An anaerobic thermophilic bacterial strain, A9 (NITE P-03545), that secretes β-glucosidase was newly isolated from wastewater sediments by screening using esculin. The 16S rRNA gene sequence of strain A9 had 100% identity with that of *Thermobrachium celere* type strain JW/YL-NZ35. The complete genome sequence of strain A9 showed 98.4% average nucleotide identity with strain JW/YL-NZ35. However, strain A9 had different physiological properties from strain JW/YL-NZ35, which cannot secrete β-glucosidases or grow on cellobiose as the sole carbon source. The key β-glucosidase gene (*TcBG1*) of strain A9, which belongs to glycoside hydrolase family 1, was characterized. Recombinant β-glucosidase (rTcBG1) hydrolyzed cellooligosaccharides to glucose effectively. Furthermore, rTcBG1 showed high thermostability (at 60°C for 2 days) and high glucose tolerance (IC_50_ = 0.75 M glucose), suggesting that rTcBG1 could be used for biological cellulose saccharification in cocultures with *Clostridium thermocellum*. High cellulose degradation was observed when strain A9 was cocultured with *C. thermocellum* in a medium containing 50 g/l crystalline cellulose, and glucose accumulation in the culture supernatant reached 35.2 g/l. In contrast, neither a monoculture of *C. thermocellum* nor coculture of *C. thermocellum* with strain JW/YL-NZ35 realized efficient cellulose degradation or high glucose accumulation. These results show that the β-glucosidase secreted by strain A9 degrades cellulose effectively in combination with *C. thermocellum* cellulosomes and has the potential to be used in a new biological cellulose saccharification process that does not require supplementation with β-glucosidases.

**Key points:**

• *Strain A9 can secrete a thermostable β-glucosidase that has high glucose tolerance*

• *A coculture of strain A9 and C. thermocellum showed high cellulose degradation*

• *Strain A9 achieves biological saccharification without addition of β-glucosidase*

**Supplementary Information:**

The online version contains supplementary material available at 10.1007/s00253-022-11818-0.

## Introduction

The bioconversion of cellulosic biomass to sugars is a major bottleneck in the development of methods for the use of cellulosic feedstocks and the commercialization of bio-based chemicals and biofuels. This bottleneck occurs because cellulosic biomass is resistant to enzymatic degradation and the hydrolytic enzymes required are expensive. β-Glucosidase is a hydrolytic enzyme that can produce glucose from cellobiose and cellooligosaccharides (Meleiro et al. 2015). Cellobiose strongly inhibits both endoglucanase and exoglucanase (Chamoli et al. 2016; Murphy et al. 2013; Zhao et al. 2013), which is considered to be the main barrier to complete cellulose degradation (Singhania et al. 2013; Sørensen et al. 2013; Teugjas and Väljamäe 2013)**.**

Many microorganisms capable of producing cellulolytic enzymes have been reported and characterized (Lynd et al. 2002). *Clostridium thermocellum (Ruminiclostridium thermocellum, Hungateiclostridium thermocellum, Acetivibrio thermocellus)*—an anaerobic thermophilic bacterium—is the most potent cellulose-degrading bacterium known to produce “cellulosomes” (Bayer et al. 2004; Lynd et al. 2002). These cellulosomes contain a large variety of enzymes, including enzymes with the potential to degrade cellulosic biomass (Bayer et al. 2004). However, feedback inhibition of cellulosomes and cellulosomal enzymes occurs as cellobiose accumulates as the final product because *C. thermocellum* is unable to produce extracellular β-glucosidases. Many efforts have been made to obtain low-cost β-glucosidases, including screening and engineering for β-glucosidases with higher activity (Gefen et al. 2012; Pei et al. 2012; Teugjas and Väljamäe 2013; Yi et al. 2013) and the development of novel processes for protein recycling (Waeonukul et al. 2012, 2013). To relieve the feedback inhibition and promote cellulose saccharification, we have demonstrated previously that glucose can be produced from cellulosic biomass by *C. thermocellum* cultures supplemented with thermostable β-glucosidases in a process called biological simultaneous enzyme production and saccharification (Prawitwong et al. 2013). Recently, Zhang *et al*. (Zhang et al. 2017) constructed a recombinant strain of *C. thermocellum* that produced an exoglucanase (named CelS) bearing heterologous β-glucosidase (BglA from *Caldicellulosiruptor* sp. F32), which was assembled in the cellulosome through a dockerin module of CelS. The production of reducing sugars from microcrystalline cellulose, such as Avicel, was enhanced using this system, which resolved the issue of feedback inhibition by hydrolyzing the accumulated cellobiose into glucose. However, to use recombinant *C. thermocellum*, it is still necessary to optimize suitable β-glucosidases for the cellulosomes. Additionally, biosafety issues must be considered when using genetically modified microbes in industrial applications (Friehs 2004; Kumar 2014).

Here, we report the newly isolated *Thermobrachium celere* strain A9. Strain A9 can secrete β-glucosidase into the culture medium. To the best of our knowledge, there are no reports on anaerobic thermophilic bacteria that can secrete appreciable amounts of β-glucosidase. Coculture of strain A9 and *C. thermocellum* has the potential to be used in the development of a new biological cellulose saccharification process that does not require any supplemental β-glucosidases or recombinant techniques.

## Materials and methods

### Materials, media, and cultivation methods

Samples of soil and wastewater sediment were collected from the layer 20 cm under the draining wastewater systems at different sampling sites at the Pilot Plant Development and Training Institute, King Mongkut’s University of Technology Thonburi, Thailand. The soil and wastewater sediment samples were stored in resealable zipper storage bags. The basal medium (BM7CO) (Waeonukul et al. 2012) was composed of (per liter) 2.9 g of K_2_HPO_4_, 1.5 g of KH_2_PO_4_, 2.1 g of urea, 6.0 g of yeast extract, 4.0 g of Na_2_CO_3_, 0.01 g of CaCl_2_·2H_2_O, 0.5 g of cysteine-HCl, 0.0005 g of resazurin, and 200 μl of mineral solution (all from FUJIFILM Wako Pure Chemicals, Osaka, Japan). BM7CO medium was degassed by boiling, then bubbled with high-purity CO_2_, and finally anaerobically distributed to Hungate tubes (Bellco Glass, Inc., Vineland, NJ, USA) and/or serum bottles. *Clostridium thermocellum* ATCC 27405^T^ was obtained from the American Type Culture Collection (ATCC) and was grown on BM7CO medium containing 10 g/l microcrystalline cellulose Sigmacell type 20 (Sigma-Aldrich; St. Louis, MO, USA). *T. celere*-type strain JW/YL-NZ35 (DSM8682^T^) was obtained from the German Collection of Microorganisms and Cell Cultures GmbH (DSMZ). Strain JW/YL-NZ35 was grown on modified DSMZ616 (YTG) medium composed of (per liter): 0.36 g of Na_2_HPO_4_·2H_2_O, 0.08 g of KCl, 5.0 g of yeast extract, 10.0 g of Bacto™ tryptone (Thermo Fisher Scientific, MA, USA), 5.3 g of Na_2_CO_3_, 0.2 g of cysteine-HCl, 0.2 g of Na_2_S, and 0.0005 g of resazurin (pH 9.0) containing 5.0 g of D-glucose or cellobiose. The YTG medium was also degassed, followed by bubbling with high-purity N_2_. *Escherichia coli* BL21 (DE3) and the plasmid pET19b (Merck KGaA, Darmstadt, Germany) served as the cloning and expression host and vector, respectively.

### Enrichment, screening, and isolation of anaerobic thermophilic bacteria secreting β-glucosidase

**Fig. 1:**
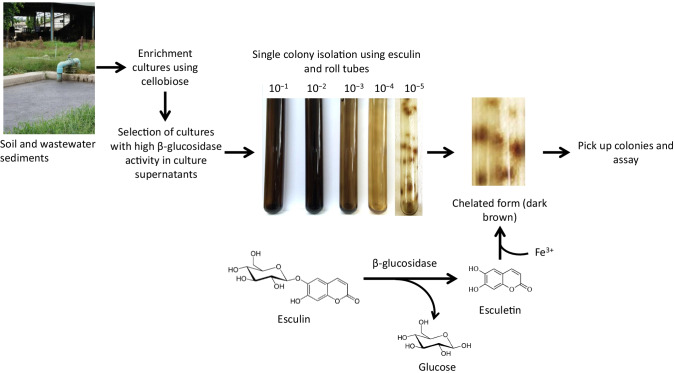
Enrichment, screening, and isolation of anaerobic thermophilic bacteria secreting β-glucosidase, using esculin. Enrichment cultures were repeated five times using BM7CO medium containing cellobiose as the sole carbon source. Single colony isolation was performed from roll tubes. Selected cultures were diluted from 10^−1^ by 10^−5^ to form colonies in roll tubes. All culturing was carried out at 60°C

Approximately 1 g of each of the collected samples of soil and wastewater sediment was inoculated directly into 10 ml of BM7CO medium containing 0.5% (w/v) cellobiose. After inoculation, the cultures were incubated at 60°C for 2–3 days. The cultures were inoculated into the same fresh medium, and the enrichment culture was repeated five times. To select cultures with high β-glucosidase activity, the supernatants of the enrichment cultures were collected by centrifugation at 10,806 × *g* at 4°C for 10 min, and the β-glucosidase activity was measured using *p*-nitrophenol-β-D-glucoside (*p*NPG; FUJIFILM Wako Pure Chemicals) as the substrate (Ahamed and Vermette 2008). Cultures that exhibited high β-glucosidase activity were selected and the enrichment culture was repeated three times using the same medium containing cellobiose. Then, single colony isolation from the cultures chosen was carried out using the Hungate roll tube technique (Hungate 1969) and BM7CO-agar medium with 5 g/l cellobiose as a carbon source; this medium was supplemented with 1 g/l esculin (Tokyo Chemical Industry Co., Ltd., Tokyo, Japan) and 2.5 g/l ferric ammonium citrate (FUJIFILM Wako Pure Chemicals). Colonies producing extracellular β-glucosidases were detected by the formation of a dark brown halo (Fig. 1). Colonies that formed a dark brown halo following degradation of esculin were individually selected from the roll tubes and inoculated into the same medium with cellobiose as described above (Fig. 1). Eventually, strain A9 was identified as the bacterium producing the highest β-glucosidase activity in culture supernatants.

### Identification and genome features of strain A9

Genomic DNA from strain A9 was extracted using a NucleoSpin® microbial DNA kit (Takara Bio, Shiga, Japan). The 16S rRNA gene was amplified using the bacterial domain-specific primers 27F and 1525R (Osborne et al. 2005). The 16S rRNA gene sequence was analyzed using the nucleotide BLAST program and manually aligned with sequences in the GenBank database using CLUSTAL_X v.1.81 (Thompson et al. 1994). A phylogenetic tree was prepared using the results of the 16S rRNA sequence comparison by the neighbor-joining method using the programs BioEdit (Hall 1999) and MEGA (Tamura et al. 2007).

Whole-genome sequence analysis of strain A9 was performed using the Ion GeneStudio S5 system, following the previously reported analytical method (Nakazono-Nagaoka et al. 2019). The genome was assembled de novo using CLC Genomics Workbench 20.0.1 (CLC Bio, Qiagen, Valencia, CA, USA). The whole-genome sequence was deposited in GenBank with accession number BMAP00000000. Average nucleotide identity (ANI) values, digital DNA–DNA hybridization (dDDH) values, and average amino acid identity (AAI) were calculated using the ANI calculator (www.ezbiocloud. Net/tools/ani) (Yoon et al. 2017), genome-to-genome distance calculator (GGDC) (http://ggdc.dsmz.de/distcalc2.php) (Meier-Kolthoff et al. 2013), and AAI calculator (http://enve-omics.ce.gatech.edu/aai) (Konstantinidis and Tiedje 2005), respectively.

### Construction of β-glucosidase expression vector and production of recombinant protein

Two oligonucleotide primers for β-glucosidase (GenBank accession number: GFR35525.1) designed from the draft genome sequence data of strain A9, 5′-GGGGATCCATGCAAAAATACACTTTCCC-3′ with a *Bam*HI site, and 5′-GGCTCAGCTCATTCACAAAGGCTATTAT-3′ with a *Bpu*1102 site, were synthesized to amplify the coding region of the β-glucosidase gene (*TcBG1*) by PCR. The PCR product was digested with *Bam*HI and *Bpu*1102, and then ligated into pET19b. The recombinant *E. coli* clone was inoculated into Luria-Bertani medium supplemented with 100 μg/ml ampicillin and incubated at 37°C. Isopropyl-β-D-thiogalactopyranoside was added to induce protein expression. The recombinant enzyme was purified using the Profinia affinity chromatography system (Bio-Rad Laboratories, Hercules, CA, USA). The homogeneity of the purified protein was determined by sodium dodecyl sulfate-polyacrylamide gel electrophoresis (SDS-PAGE; ATTO, Tokyo, Japan). Molecular mass standards were from Bio-Rad Laboratories.

### Enzyme activity and zymogram analysis

β-Glucosidase activity was determined by measuring the hydrolysis of *p*NPG. The reaction mixture contained 10 μl of enzyme and 100 μl of 1 mM *p*NPG in 100 mM sodium acetate buffer, pH 6.0. After incubation at 60°C for 10 min, the reaction was stopped by adding 200 μl of 0.4 M Na_2_CO_3_. The released *p*-nitrophenol was measured using a spectrophotometer at 405 nm (Waeonukul et al. 2012). One unit (U) of β-glucosidase activity was defined as the amount of enzyme that liberated 1 μmol of *p*-nitrophenol per min per ml. The activity of the enzyme toward cellobiose and cellooligosaccharides (cellotriose, cellotetraose, cellopentaose, and cellohexaose; Megazyme, Bray, Ireland) was also assayed at 60°C for 10 min. The reaction mixture contained 50 μl of enzyme and 50 μl of 50 mg/ml cellooligosaccharide in 100 mM sodium acetate buffer, pH 6.0. The concentration of glucose released from the cellooligosaccharides was determined using a glucose oxidase–peroxidase assay kit (FUJIFILM Wako Pure Chemicals). One U of enzyme activity was defined as the amount of enzyme that liberated 1 μmol of glucose per min per ml. To determine the mode of action toward cellooligosaccharides, reaction mixtures were collected after 10, 20, 30, 40, 50 and 60 s. Each substrate (10 mg/ml) was incubated with enzyme (0.5 U) at 60°C. The hydrolysis products were analyzed using thin-layer chromatography. Protein concentrations were determined using a Coomassie protein assay kit (Thermo Fisher Scientific, Waltham, MA, USA) with bovine serum albumin as the standard.

The optimum pH for enzyme activity was determined at 60°C with *p*NPG as substrate in buffers with pH values 4.0–9.0. The buffers used were 100 mM sodium acetate for pH 4.0–6.0, 100 mM phosphate for pH 6.0–8.0, and 100 mM Tris-HCl for pH 8.0–9.0. The optimal temperature for enzyme activity was determined at pH 6.0 (in sodium acetate buffer) from 40 to 80°C. Thermostability was determined by preincubating the enzyme (1 μg protein) without substrate in sodium acetate buffer (pH 6.0) for 1 h at 50–80°C; the residual β-glucosidase activity for each condition was then assayed at 60°C to determine thermostability. Also, enzyme in sodium acetate buffer (pH 6.0) was incubated at 60°C for 7 days; a sample of the incubated enzyme was collected every 24 h to measure the residual β-glucosidase activity. Glucose inhibition of β-glucosidase activity was measured by adding glucose at different concentrations (0–1.0 M) to the standard reaction mixture with *p*NPG as the substrate. The glucose concentration required to inhibit 50% of the initial β-glucosidase activity (the IC_50_ value) was determined. The relative activity (%) was defined as the value relative to the activity in the absence of glucose.

Native PAGE was performed at pH 8.3 using 10% acrylamide as the resolving gel with a 5% stacking gel (pH 6.8). After electrophoresis, the gel was soaked in 100 mM sodium acetate buffer (pH 6.0) for 10 min at room temperature. β-Glucosidase activity in the gel was detected by staining with esculin mixed with ferric ammonium citrate or using 4-methylumbelliferyl-β-D-glucoside (4-MUG; FUJIFILM Wako Pure Chemicals). For staining with esculin mixed with ferric ammonium citrate, the gel was incubated in 100 mM sodium acetate buffer containing 1 g/l esculin and 2.5 g/l ferric ammonium citrate for 10–30 min at 50°C (Kwon et al. 1994). For the detection of β-glucosidase using 4-MUG, the gel was incubated in 100 mM sodium acetate buffer containing 10 mM 4-MUG for 30 min at 50°C. The active bands in the gel were detected under UV light using a gel documentation system.

### Monitoring of bacterial growth and extracellular β-glucosidase production

Strain A9 and *T. celere-*type strain JW/YL-NZ35 were cultured in BM7CO and YTG medium, respectively, containing 5 g/l cellobiose or other carbohydrates (e.g., xylose and arabinose). In the YTG medium, cellobiose or other carbohydrates were used as carbon sources instead of glucose. Cell growth was measured by cell turbidity determined using a spectrophotometer at 600 nm (Thermo Fisher Scientific) and 10-mm pathlength cuvettes. The total cell growth was determined by measuring the total protein concentration following a previous report (Shikata et al. 2018). The culture supernatants during cell growth were collected and analyzed for β-glucosidase activity using zymography.

Cells of strain A9 during the exponential phase were observed by scanning electron microscopy (SEM; Jeol JSM-6320F, Tokyo, Japan) in accordance with the manufacturer’s instructions.

### Saccharification of microcrystalline cellulose by coculture of strain A9 and *C. thermocellum*

For subculturing, *C. thermocellum* ATCC27405^T^ stock culture was inoculated by syringe into 5 mL of BM7CO medium containing 10 g/l cellulose and was incubated at 60°C in anaerobic conditions. The subculture of *C. thermocellum* ATCC27405^T^ was inoculated again by syringe into BM7CO medium containing 50 g/l Sigmacell type 20 at 60°C for 2 days. To avoid the possibility of competitive growth inhibition of *C. thermocellum* in the coculture, the subculture of strain A9 or *T. celere-*type strain JW/YL-NZ35 was inoculated after cultivation of *C. thermocellum* for 2 days. The cocultures of *C. thermocellum* and strain A9 or type strain JW/YL-NZ35 were incubated at 60°C for 10 days in anaerobic conditions. The β-glucosidase activity and the concentration of accumulated glucose in the culture supernatants were monitored by an enzymatic assay using *p*NPG and high-performance liquid chromatography (Shimadzu Corp., Kyoto, Japan), respectively.

## Repositories

### National Institute of Technology and Evaluation (NITE): KM-A9 (NITE P-03545)

The GenBank accession numbers for the genome sequences of *Thermobrachium celere* strain A9 and *T. celere* type strain JW/YL-NZ35 are BMAP00000000 and CAVN000000000, respectively.

## Results

### Enrichment, screening, and isolation of anaerobic thermophilic bacteria producing extracellular β-glucosidases

More than 100 samples of soil and wastewater sediment were collected and individually cultured in BM7CO medium containing 5 g/l cellobiose as the sole carbon source in thermophilic growth conditions (i.e., 60°C). The cultures grew well on the cellobiose medium. To isolate bacteria that produced high β-glucosidase activity in the culture supernatants, enrichment culturing was performed at least five times. Single colonies were separated by the anaerobic Hungate roll tube technique using cellobiose as the sole carbon source in an agar medium supplemented with esculin and ferric ammonium citrate. Generally, *p*NPG and 4-MUG are used to screen for microbes producing β-glucosidase; however, these chemicals are unstable over long periods of incubation at >50°C, and, therefore, they are not suitable for use as substrates for the screening of thermophilic microbes producing β-glucosidase. Thus, esculin, which is stable during incubation at high temperatures, was used as the substrate. The principle of the assay is the formation of a dark brown color, which is a result of the chelation of esculetin, produced from esculin by β-glucosidase, with ferric ions in the medium (Fig. 1). Therefore, it is expected that bacteria that secrete β-glucosidase will show a dark brown halo around their colonies (Fig. 1). A dark brown halo was observed around many of the colonies in the roll tubes (Fig. 1). Colonies that formed a dark brown halo were picked up from the roll tubes and inoculated again into BM7CO liquid medium containing cellobiose. To confirm whether these colonies could secrete β-glucosidase into the culture medium, the β-glucosidase activity in the supernatants from each culture was measured. Approximately 20 cultures were observed to have relatively high β-glucosidase activity in the culture supernatant; strain A9 showed the highest β-glucosidase activity of all the cultures. To confirm the bacterial purity, strain A9 was verified by repeated single colony isolation from roll tube cultures and its morphology was investigated using microscopic analysis.

**Fig. 2:**
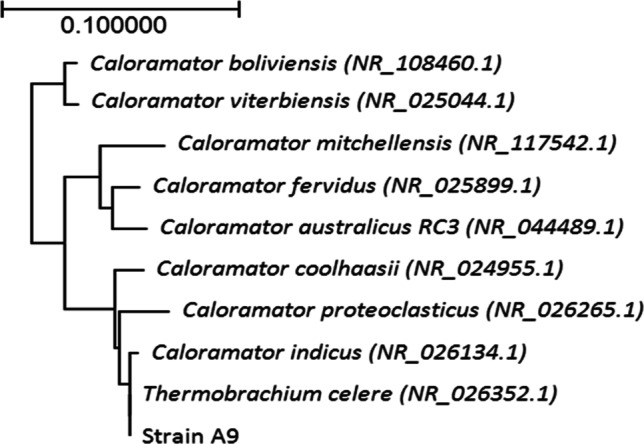
Neighbor-joining phylogenetic tree generated using 16S rRNA gene sequences. The tree was constructed using a distance-matrix analysis of 16S rRNA gene sequences. Bootstrap percentages were obtained from 1000 resamplings. The bar represents 0.100 nucleotide changes per sequence position

The full-length 16S rRNA gene sequence of the isolated strain A9 (GenBank accession number: OK_036794.1) was compared with other sequences from the GenBank database. Homology and phylogenetic analyses of sequences showed that the 16S rRNA gene of strain A9 had 100% identity with that of *T. celere* type strain JW/YL-NZ35 (DSM8682^T^) (Engle et al. 1996) (GenBank accession number: NR_026352.1), 99.0% identity with that of *Caloramator indicus* strain IndiB4 (Chrisostomos et al. 1996) (GenBank accession number: NR_026134.1), and 98.0% identity with that of *Calo. coolhaasii* strain Z (Plugge et al. 2000) (Fig. 2). The most similar bacterium, *T. celere* type strain JW/YL-NZ35, isolated from hot spring sediments in New Zealand, has been reported to produce hydrogen at high yields, and can produce an appreciable amount of ethanol depending on the growth conditions (Ciranna et al. 2011, 2012; Engle et al. 1996).

The whole-genome sequence of strain A9 was deposited in GenBank with accession number BMAP00000000. ANI values, dDDH values, and AAI were calculated between strain A9 and *T. celere* type strain JW/YL-NZ35. The ANI and dDDH values, which were based on the genomic sequences of strain A9 and type strain JW/YL-NZ35 (GenBank accession no. CAVN000000000), were 98.4% and 85.8%, respectively; these are above the defined thresholds for species delineation of 95–96% for ANI and 70% for GGDC (Goris et al. 2007; Richter and Rosselló-Móra 2009). *T. celere* strain A9 was deposited as KM-A9 (NITE P-03545) in the National Institute of Technology and Evaluation (NITE), Chiba, Japan.

*T. celere* is also known as *Caloramator celer* (Baena and Patel 2009; Ciranna et al. 2013); however, there is clear taxonomic reason for using this alternative name*.* When type strain *T. celere* JW/YL-NZ35 was isolated by Engle *et al*., there was a known relationship between *T. celere* and *Calo. fervidus*, which is formally named *Clostridium fervidus* (Patel et al. 1987)*,* from 16S rRNA sequence analysis; however, there was no mention of *Calo. celer*. Additionally, type strain JW/YL-NZ35 is still listed as *T. celere* in the List of Prokaryotic Names with Standing in Nomenclature (https://lpsn.dsmz.de/species/thermobrachium-celere) and BacDive (https://bacdive.dsmz.de/strain/2516), which are popular worldwide databases for standardized bacterial information. We have used *T. celere* as the species name of strain A9 in this report to avoid confusion between genera.

### Characterization of extracellular β-glucosidase from strain A9

**Fig. 3:**
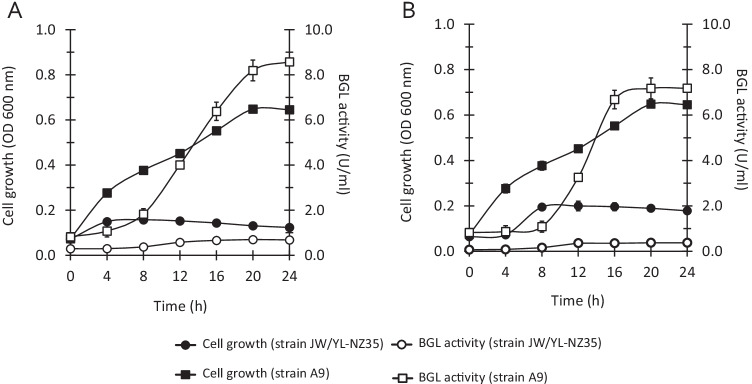
Time course of cell growth and extracellular β-glucosidase (BGL) activity of strain A9 and *Thermobrachium celere* type strain JW/YL-NZ35 grown on media containing cellobiose (A) or glucose (B) as carbon sources, respectively. BM7CO and YTG media were used as the basal media for strain A9 and type strain JW/YL-NZ35, respectively. The data are the means of three independent experiments. Error bars represent the standard deviation (*n* = 3)

To determine whether strain A9 could produce and secrete β-glucosidase into the culture medium during growth, the cell growth and extracellular β-glucosidase activity of strain A9 in BM7CO medium containing cellobiose as a carbon source were monitored (Fig. 3A). β-Glucosidase activity of 1.0 U/ml was detected in the culture medium after 4 h and this increased to 8.5 U/ml (8.29 U/mg protein) after 20 h (Fig. 3A). In contrast, no growth or extracellular β-glucosidase activity of *T. celere* type strain JW/YL-NZ35 was observed in cellobiose-containing medium based on YTG medium (Fig. 3A). One study reported that type strain JW/YL-NZ35 cannot use cellobiose as a carbon source (Engle et al. 1996). Strain JW/YL-NZ35 grew on the glucose medium; however, no extracellular β-glucosidase activity was observed in the culture medium (Fig. 3B). In contrast, in the glucose medium, the β-glucosidase activity and cell density of strain A9 were 7.17 U/ml (8.07 U/mg protein) and OD_600 nm_ = 0.51, respectively, which are similar values to when strain A9 was grown on cellobiose medium (Fig. 3A). These results suggest that the expression of extracellular β-glucosidase by strain A9 may not be affected by the carbon source present in the medium.

**Fig. 4:**
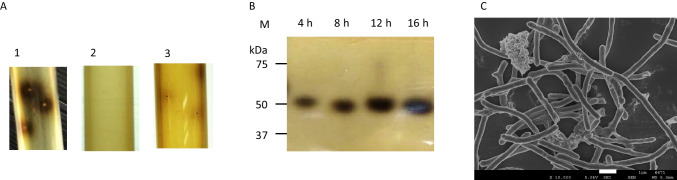
Confirmation of β-glucosidase activity using esculin (A, B) and morphology (C) of strain A9. β-Glucosidase activity in the culture supernatants of strain A9 and *T. celere* type strain JW/YL-NZ35 was confirmed by roll tube (A) and zymogram analysis (B) using esculin. A-1: Colonies of strain A9 on BM7CO medium containing 0.5% cellobiose. A-2: Colony formation of type strain JW/YL-NZ35 could not be observed on YTG medium containing 0.5% cellobiose. A-3: Colonies of type strain JW/YL-NZ35 on YTG medium containing 0.5% glucose. (B) Lane M, standard protein molecular weight markers (kDa). (C) Scanning electron microscopy image showing the morphology of strain A9 after cultivation for 16 h. Scale bar, 1 μm

To visualize the presence of β-glucosidases in the culture medium, colonies in roll tubes were analyzed using staining with esculin and ferric ammonium citrate, and culture supernatants of strain A9 during the logarithmic (4 h of cultivation) to stationary (16 h of cultivation) phases were analyzed for β-glucosidase activity by zymography with the same stain (Fig. 4A and B). A large, dark halo surrounded colonies of strain A9, indicating that this strain can grow in cellobiose medium and secrete β-glucosidase (Fig. 4A-1). Meanwhile, no colonies of *T. celere* type strain JW/YL-NZ35 were observed on YTG-agar containing cellobiose (Fig. 4A-2), and no halos were observed around colonies of *T. celere* type strain JW/YL-NZ35 grown on glucose medium (Fig. 4A-3). Zymogram analysis clearly showed the presence of secreted β-glucosidase (Fig. 4B) in the culture supernatants of strain A9, and the activity increased with cultivation time. To investigate whether strain A9 produced and secreted β-glucosidase from cells without cell lysis, cells of strain A9 during the exponential phase (16 h of cultivation) were imaged by SEM. The SEM results showed that the cells of strain A9 had a complete structure and did not show lysis or any other disruption (Fig. 4C). These data indicate that strain A9 secretes β-glucosidase into the culture medium without cell lysis.

### Characterization of β-glucosidase (TcBG1) from strain A9

According to draft genome sequence analysis, strain A9 and *T. celere* type strain JW/YL-NZ35 have predicted β-glucosidases belonging to glycoside hydrolase (GH) families 1 (GenBank accession number: GFR35525.1) and 30 (GenBank accession number: GFR35524.1), and GH families 1 (GenBank accession number: CDF59168.1), 3 (GenBank accession number: CDF57462.1), and 30 (GenBank accession number: CDF59169.1), respectively. However, β-glucosidase activity is primarily found in GH families 1 and 3, and is less common in GH family 30 (Salgado et al. 2018). β-Glucosidases that belong to GH family 3 are strongly inhibited at low glucose concentrations. In contrast, most enzymes of GH family 1 show tolerance to glucose and have higher hydrolytic activity toward cellobiose than enzymes in GH family 3 (Srivastava et al. 2019). To understand the properties of the extracellular β-glucosidase from strain A9, we characterized the β-glucosidase (GFR35525.1) that had a molecular mass of approximately 50 kDa by zymogram analysis (Fig. 4B). The gene encoding this β-glucosidase was cloned and recombinant protein was produced.

**Fig. 5:**
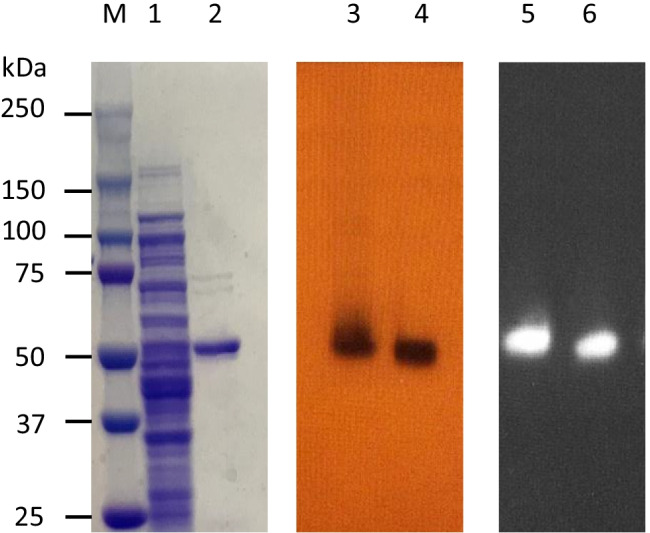
SDS-PAGE and zymogram analyses of recombinant β-glucosidase from strain A9 expressed in *Escherichia coli* (rTcBG1). Lane M, standard protein molecular weight markers. Lane 1, crude extract from recombinant *E. coli*-expressing rTcBG1; Lane 2, purified rTcBG1; Lanes 3 and 4, and 5 and 6, zymogram analysis of rTcBG1 and culture supernatant of strain A9 stained by esculin mixed with ferric ammonium citrate (lanes 3 & 4) or 4-methylumbelliferyl-β-D-glucoside (lanes 5 & 6)

**Fig. 6:**
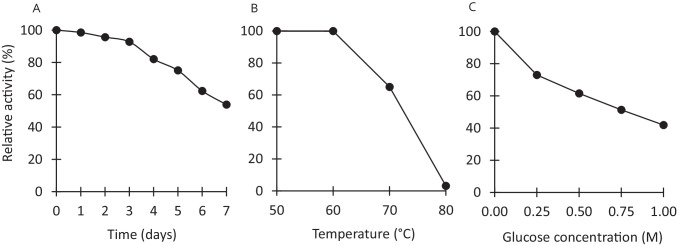
Enzymatic characterization of rTcBG1. rTcBG1 was purified and its thermostability and glucose tolerance were determined. The stability of rTcBG1 was measured at 60 °C over time (A) and at different temperatures after incubation for 1 h (B). The glucose tolerance of rTcBG1 was measured at different glucose concentrations (C). The activity of the enzyme in the absence of glucose was defined as 100%. The data are the means of three independent experiments. Error bars represent the standard deviation (<0.35%)

**Fig. 7:**
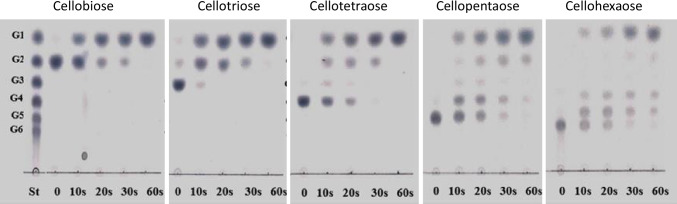
Thin-layer chromatography of the hydrolysis products from cellooligosaccharides hydrolyzed by rTcBG1. Glucose (G1), cellobiose (G2), cellotriose (G3), cellotetraose (G4), cellopentaose (G5), and cellohexaose (G6) were used as standards (shown at the left-hand side)

The nucleotide sequence of the β-glucosidase gene (*TcBG1*) was 1,347 bp, encoding 448 amino acids with a calculated protein mass of 52 kDa. In SDS-PAGE analysis, the molecular mass of the recombinant β-glucosidase (rTcBG1) (Fig. 5-1 and -2) agreed with the molecular mass of the native β-glucosidase of strain A9 (Figs. 4B and 5-2). Zymogram analysis (Fig. 5-3, -4 and -5, -6) of rTcBG1 and the native β-glucosidase showed dark brown and white bands from the hydrolysis of esculin and 4-MUG, respectively. rTcBG1 exhibited its maximum enzymatic activity at pH 6.0–7.0 and 60–70°C. rTcBG1 was stable at 60°C for 72 h, retaining almost 100% of the initial activity (half-life 168 h) (Fig. 6A); after incubation at 70°C for 1 h, rTcBG1 retained 65% activity, but after incubation at 80°C for 1 h, >90% of the activity was lost (Fig. 6B). rTcBG1 retained 50% of the initial activity in the presence of a high glucose concentration (1 M) (Fig. 6C), which suggested that the extracellular β-glucosidase of strain A9 had a higher tolerance to glucose than GH1 β-glucosidases from other thermophilic anaerobic bacteria (Table 1). rTcBG1 also exhibited specific activity toward the cellooligosaccharides cellobiose, cellotriose, cellotetraose, cellopentaose, and cellohexaose with activities of 49.8, 47.6, 43.6, 30.7, and 28.8 U/mg protein, respectively (Table 2), suggesting that rTcBG1 can hydrolyze short-chain cellooligosaccharides faster than long-chain cellooligosaccharides (Fig. 7). These results for rTcBG1 indicate that the extracellular β-glucosidase of strain A9 has favorable properties as a supplemental enzyme source for the biological saccharification of high concentrations of cellulose using *C. thermocellum*.

**Table 1:** Characterization of rTcBG1 from strain A9 compared with that of family GH1 β-glucosidases from other anaerobic thermophilic bacteria

Enzyme	Optimum temperature for reaction (°C)^a^	Thermostability (%)^b^	Glucose inhibition: IC_50_ (M)^c^	Reference
TcBG1 (*Thermobrachium celere* strain A9)	60–70	99	0.75 ± 0.01	This study
CglT (*Thermoanaerobacter brokii*)	60–70	97	0.45 ± 0.01	Waeonukul et al. (2012)
Bgl (*Thermoanaerobacterium thermosaccharolyticum* Noi-1)	60–70	10	0.65 ± 0.01	Prawitwong et al. (2013)
BgIA (*Clostridium thermocellum* ATCC27405^T^)	50–60	10	0.40 ± 0.01	Prawitwong et al. (2013)

^a^The range reported is where >90% of the maximal activity was maintained

^b^Thermostability was determined as the percentage of remaining β-glucosidase activity after incubation of the enzyme at 60°C for 24 h

^c^The glucose concentration required to inhibit 50% of the initial β-glucosidase activity. Results for glucose inhibition are provided as the mean ± standard deviation (*n* = 3)

**Table 2:** Specific activity of rTcBG1 from strain A9 toward cellooligosaccharides

Substrate	Specific activity (U/mg protein)^a^
Cellobiose	49.78 ± 0.15
Cellotriose	47.60 ± 0.25
Cellotetraose	43.61 ± 0.26
Cellopentaose	30.68 ± 0.18
Cellohexaose	28.76 ± 0.58

^a^One unit (U) of enzyme activity was defined as the amount of enzyme that liberated 1 μmol of glucose in 1 min. Results are the mean ± standard deviation (*n* = 3)

### Cellulose saccharification using a coculture of strain A9 and *C. thermocellum*

**Fig. 8:**
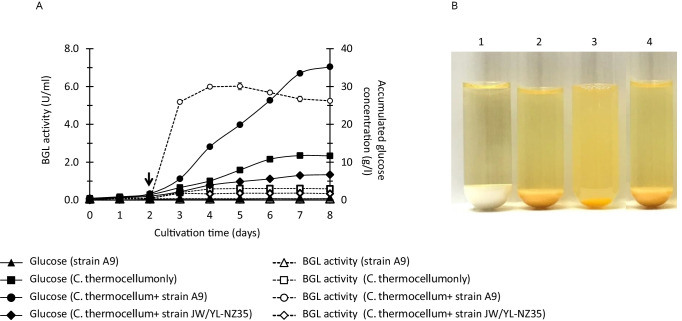
Cellulose saccharification using cocultures of strain A9 or *T. celere* type strain JW/YL-NZ35 with *C. thermocellum* ATCC27405^T^. The glucose concentration and β-glucosidase activity in the culture supernatants were measured by high-performance liquid chromatography and by using *p*NPG, respectively (A). The arrow indicates the inoculation point of strain A9 or type strain JW/YL-NZ35. Cellulose saccharification by cocultures shown in cultivation pictures taken after 8 days (B). Lane 1, BM7CO containing 5% (w/v) cellulose inoculated with strain A9; lane 2, BM7CO containing 5% (w/v) cellulose inoculated with *C. thermocellum* only; lane 3, BM7CO containing 5% (w/v) cellulose inoculated with strain A9 and *C. thermocellum*; lane 4, BM7CO containing 5% (w/v) cellulose inoculated with type strain JW/YL-NZ35 and *C. thermocellum.* The data are the means of three independent experiments. Error bars represent the standard deviation (*n* = 3)

Studies have demonstrated that the cellulose degradation ability is drastically enhanced, and glucose is accumulated in the culture medium, when *C. thermocellum* cultures are supplemented with thermostable β-glucosidases (Gefen et al. 2012; Prawitwong et al. 2013). This enhancement occurs because feedback inhibition caused by cellobiose can be resolved by supplementation with extracellular β-glucosidases. To evaluate whether strain A9 can replace this supplementation with β-glucosidase, a coculture of strain A9 and *C. thermocellum* was investigated using a medium containing a high concentration of microcrystalline cellulose (Fig. 8). When strain A9 was cocultured with *C. thermocellum* in BM7CO medium containing 50 g/l microcrystalline cellulose, cellulose was effectively converted to glucose, and a high concentration of glucose accumulated in the culture supernatant. The saccharification profile of the coculture of strain A9 and *C. thermocellum* showed efficient cellulose degradation, and glucose accumulation was increased after 3 days. The cellulose was degraded almost entirely within 8 days and the glucose accumulation reached 35.2 g/l in the culture supernatant (70.4% of the theoretical glucose yield based on the loading of microcrystalline cellulose) (Fig. 8A and B), which was almost the same yield as that obtained with purified β-glucosidase supplementation to culture of *C. thermocellum* (Prawitwong et al. 2013).

In contrast, monoculture of *C. thermocellum* or coculture of *C. thermocellum* with *T. celere* type strain JW/YL-NZ35 did not efficiently hydrolyze cellulose and had low β-glucosidase activity (Fig. 8A and B). The accumulated glucose concentration was <11.7 g/l (23.4% glucose yield), and cellulose substrate remained in the culture medium after 8 days (Fig. 8A, B). Strain A9 alone could not be grown in the cellulose medium (Fig. 8A and B). These results indicate that the extracellular β-glucosidase of strain A9 can cooperate with *C. thermocellum* cellulosomes and efficiently resolve feedback inhibition of the cellulosomes by cellobiose. Thus, strain A9 may be useful in combination with *C. thermocellum* to achieve biological cellulose saccharification without any enzyme supplementation.

## Discussion

When *C. thermocellum* is grown on a medium containing a high cellulose concentration supplemented with a thermostable β-glucosidase, glucose is accumulated as an end-product at a high concentration in the culture supernatant. This direct saccharification of cellulose may result from resolution of the feedback inhibition of cellulosomes and cellulases by cellobiose, and the low affinity glucose uptake and the kinetics of cellobiose and cellooligosaccharide use by *C. thermocellum* (Nataf et al. 2009; Strobel et al. 1995; Zhang and Lynd 2005). The high cellulose degradation ability and high glucose accumulation capacity of the combination of *C. thermocellum* and β-glucosidase are promising as a direct cellulose saccharification technology without the need for supplementation of any cellulases or hemicellulases. Recently, a method to avoid the extra addition of β-glucosidases, using a recombinant *C. thermocellum* that can secrete β-glucosidase, has been developed (Qi et al. 2021; Zhang et al. 2017). This method of cellulose saccharification, termed whole-cell catalysis, is a useful technology for supplementation with β-glucosidases.

The complexity of biomasses with components such as hemicellulose means that it may be challenging to promote efficient degradation using recombinant *C. thermocellum* alone. Compounds such as xylooligosaccharides have been shown to cause a reduction in cellulolytic activity and a reduction in the maximum saccharification rate of crystalline cellulose (Beri et al. 2021). Cocultures of *C. thermocellum* with hemicellulose-consuming microbes using high concentrations of corn fiber exhibited lower levels of unfermented hemicellulose hydrolysis products and the maximum saccharification rate was increased, compared with culture of *C. thermocellum* alone (Beri et al. 2021). Strain A9 can use pentoses, such as xylose, arabinose, and xylan (Table S1). According to the draft genome sequence, strain A9 and *T. celere* type strain JW/YL-NZ35 possess an enzyme from GH family 30 (GenBank accession numbers: GFR35524.1 and CDF59169.1), which is a relatively novel xylanase that can be exo-acting and liberates xylose and xylobiose from the non-reducing end of xylan (Puchart et al. 2021). Type strain JW/YL-NZ35 could not use xylose or xylan (Table S1). However, strain A9 may be an excellent partner for coculture with thermophilic microbes for biological saccharification. Thus, it is desirable to develop not only *C. thermocellum* metabolically engineered by recombinant techniques, but also microbes that are applicable in coculture systems.

To develop an efficient saccharification system using natural biomass for practical applications, it is necessary to investigate anaerobic thermophilic microbes that can secrete β-glucosidases into the culture supernatant to work in cooperation with cellulosomes and cellulases. To date, few reports have investigated the screening and isolation of aerobic microbes that can produce β-glucosidase (Ahamed and Vermette 2008). Most anaerobic thermophilic bacteria possess intracellular β-glucosidases (Aït et al. 1982; Katayeva et al. 1992; Yang et al. 2015). One study has reported that *C. stercorarium* can partially secrete β-glucosidase into the culture medium when grown on cellobiose; however, most of the β-glucosidase activity remained cell-bound, with secretion only of a low quantity of β-glucosidase into the medium (Bronnenmeier and Staudenbauer 1988). To the best of our knowledge, the present study is the first report of the isolation of an anaerobic thermophilic bacterium capable of secreting β-glucosidase. The presence of a signal sequence is essential for the secretion of enzymes outside of the cell wall. According to the signal peptide predictor tool SignalP-5.0 (http://www.cbs.dtu.dk/services/SignalP/), the β-glucosidases (GFR35525.1 and CDF59168.1) of strain A9 and *T. celere* type strain JW/YL-NZ35 have a signal peptide sequence at the *N*-terminus; however, at least one other β-glucosidase listed in Table 1 does not have this feature. The β-glucosidases of strains A9 and JW/YL-NZ35 were 100% identical in amino acid sequence. Strain A9 may have more useful properties for cellobiose culture and β-glucosidase production than type strain JW/YL-NZ35. Type strain JW/YL-NZ35 has shown only poor β-glucosidase activity and low growth on cellobiose medium, even though JW/YL-NZ35 possesses the same GH family 1 β-glucosidase gene with the same amino acid sequence as strain A9. It is still unclear what the differences are between strain A9 and type strain JW/YL-NZ35; however, further characterization of strain A9 may provide more information regarding the expression and control of endogenous β-glucosidases in anaerobic microbes.

According to the Carbohydrate-Active Enzymes database (CAZy; http://www.cazy.org), β-glucosidases are mainly found in GH families 1 and 3 (Singh et al. 2016). However, this classification based on amino acid sequence similarity and conserved motifs does not consider many important functional aspects of β-glucosidases, such as glucose tolerance and glucose stimulation. Recently, categorization of β-glucosidases based on the effect of glucose on catalytic activity has been proposed (Cao et al. 2015; Srivastava et al. 2019). This functional classification divides β-glucosidases into four classes: (I) β-glucosidases that are strongly inhibited by low concentrations of glucose; (II) β-glucosidases that are glucose tolerant; (III) β-glucosidases that are stimulated by low concentrations of glucose and inhibited by high concentrations of glucose; and (IV) β-glucosidases that are not inhibited by high glucose concentration. The β-glucosidases from strain A9 and other anaerobic thermophilic bacteria (Table 1) may belong to class II (Cao et al. 2015; Salgado et al. 2018). Most of the β-glucosidases categorized as class II that have been characterized to date belong to GH family 1 and they have been isolated from a wide variety of sources, including bacteria, fungi, and from screening metagenomic DNA libraries (Salgado et al. 2018).

The maximum β-glucosidase activity of strain A9 was 6.02 ± 0.18 U/ml (17.7 ± 0.2 U/mg protein) when cocultured with *C. thermocellum*, while the production of extracellular β-glucosidase by recombinant *C. thermocellum* was 4.91 ± 0.44 U/ml in medium containing 50 g cellulose/l (Qi et al. 2021). It is desirable to determine whether the extracellular β-glucosidase activity of strain A9 is sufficient for the complete degradation of cellulose at high concentrations (such as 100 g cellulose/l). β-Glucosidase activity of 30–50 U/g cellulose was necessary to completely degrade 100 g/l cellulose when *C. thermocellum* S14 was cultured with the addition of CglT (Table 1) (Prawitwong et al. 2013). If strain A9 can consistently produce 6 U/ml of β-glucosidase in coculture with *C. thermocellum* in medium containing 100 g/l cellulose, the β-glucosidase activity will reach 60 U/g cellulose; if this activity can be achieved, coculture with strain A9 may be able to provide sufficient β-glucosidase activity for the complete degradation of 100 g/l cellulose. A β-glucosidase secreted from recombinant *C. thermocellum* [a different enzyme (BglA of *Caldicellulosiruptor* sp. F32) (Qi et al. 2021) than those produced by strain A9 and CglT (Table 1)], had sufficient β-glucosidase activity in saccharification tests using 50 g/l microcrystalline cellulose to degrade an estimated 50 U/g glucan. Although higher β-glucosidase activity than that of BglA might be obtained if the gene from strain A9 were used in recombinant *C. thermocellum*, supplementation of recombinant BglA with >50 U of β-glucosidase resulted in no further enhancement of glucose accumulation (Qi et al. 2021). Using strain A9 can relieve the inhibitory effect of cellobiose on cellulosomes during the biological saccharification process. Thus, coculture of strain A9 and *C. thermocellum* has potential in the development of a new biological cellulose saccharification process that does not require supplementation with external β-glucosidases.

## Supplementary Information

Below is the link to the electronic supplementary material.

ESM1 (PDF 129 KB)

## Data Availability

The datasets generated and analyzed during the current study are available in the GenBank repository. Accession numbers for the genome sequence of *Thermobrachium celere* strain A9 and *T. celere* type strain JW/YL-NZ35 are BMAP00000000 [https://www.ncbi.nlm.nih.gov/nuccore/BMAP00000000.1/] and CAVN000000000 [https://www.ncbi.nlm.nih.gov/nuccore/CAVN000000000.1/], respectively.

## References

[CR1] Ahamed A, Vermette P (2008). Enhanced enzyme production from mixed cultures of *Trichoderma reesei* RUT-C30 and *Aspergillus niger* LMA grown as fed batch in a stirred tank bioreactor. Biochem Eng J.

[CR2] Aït N, Creuzet N, Cattaneo J (1982). Properties of β-Glucosidase Purified from *Clostridium thermocellum*. Microbiology.

[CR3] Baena S, Patel BK. 2009. Genus V. Caloramator. In Paul DV. (ed), Bergey's manual of systematic bacteriology, 2nd ed, vol 3 The Firmicutes Springer-Verlag, New York, NY p 834–838

[CR4] Bayer EA, Belaich JP, Shoham Y, Lamed R (2004). The cellulosomes: multienzyme machines for degradation of plant cell wall polysaccharides. Annu Rev Microbiol.

[CR5] Beri D, Herring CD, Blahova S, Poudel S, Giannone RJ, Hettich RL, Lynd LR (2021). Coculture with hemicellulose-fermenting microbes reverses inhibition of corn fiber solubilization by *Clostridium thermocellum* at elevated solids loadings. Biotechnol Biofuels.

[CR6] Bronnenmeier K, Staudenbauer WL (1988). Purification and properties of an extracellular β-glucosidase from the cellulolytic thermophile *Clostridium stercorarium*. Appl Microbiol Biotechnol.

[CR7] Cao LC, Wang ZJ, Ren GH, Kong W, Li L, Xie W, Liu YH (2015). Engineering a novel glucose-tolerant β-glucosidase as supplementation to enhance the hydrolysis of sugarcane bagasse at high glucose concentration. Biotechnol Biofuels.

[CR8] Chamoli S, Kumar P, Navani NK, Verma AK (2016). Secretory expression, characterization and docking study of glucose-tolerant β-glucosidase from *B. subtilis*. Int J Biol Macromol.

[CR9] Chrisostomos S, Patel BKC, Dwivedi PP, Denman SE (1996). Caloramator indicus sp. nov., a new thermophilic anaerobic bacterium isolated from the deep-seated nonvolcanically heated waters of an indian artesian aquifer. Int J Syst Evol Micr.

[CR10] Ciranna A, Larjo A, Kivistö A, Santala V, Roos C, Karp M (2013). Draft genome sequence of the hydrogen- and ethanol-producing anaerobic alkalithermophilic bacterium *Caloramator celer*. Genome Announc.

[CR11] Ciranna A, Santala V, Karp M (2011). Biohydrogen production in alkalithermophilic conditions: *Thermobrachium celere* as a case study. Bioresour Technol.

[CR12] Ciranna A, Santala V, Karp M (2012). Enhancing biohydrogen production of the alkalithermophile *Thermobrachium celere*. Int J Hydrogen Energ.

[CR13] Engle M, Li Y, Rainey F, Deblois S, Mai V, Reichert A, Mayer F, Messner P, Wiegel J (1996) *Thermobrachium celere* gen. nov., sp. nov., a rapidly growing thermophilic, alkalitolerant, and proteolytic obligate anaerobe. Int J Syst Evol Microbiol 46(4):1025–1033 10.1099/00207713-46-4-1025 10.1099/00207713-46-4-1025 8863432

[CR14] Friehs K (2004). Plasmid copy number and plasmid stability. Adv Biochem Eng Biotechnol.

[CR15] Gefen G, Anbar M, Morag E, Lamed R, Bayer EA (2012). Enhanced cellulose degradation by targeted integration of a cohesin-fused β-glucosidase into the *Clostridium thermocellum* cellulosome. Proc Natl Acad Sci USA.

[CR16] Goris J, Konstantinidis KT, Klappenbach JA, Coenye T, Vandamme P, Tiedje JM (2007). DNA-DNA hybridization values and their relationship to whole-genome sequence similarities. Int J Syst Evol Microbiol.

[CR17] Hall TA (1999). BioEdit : a user-friendly biological sequence alignment editor and analysis program for Windows 95/98/NT. Nucleic Acids Symp Ser.

[CR18] Hungate RE (1969) Chapter IV A roll tube method for cultivation of strict anaerobes. In: Norris JR, Ribbons DW (eds) Methods Microbiol. vol 3. Academic Press Inc. London, pp117–132. 10.1016/S0580-9517(08)70503-8

[CR19] Katayeva IA, Golovchenko NP, Chuvilskaya NA, Akimenko VK (1992). *Clostridium thermocellum* β-glucosidases A and B: purification, properties, localization, and regulation of biosynthesis. Enzyme Microb Technol.

[CR20] Konstantinidis KT, Tiedje JM (2005). Towards a genome-based taxonomy for prokaryotes. J Bacteriol.

[CR21] Kumar S (2014). Biosafety issues of genetically modified organisms. Biosafety.

[CR22] Kwon KS, Lee J, Kang HG, Hah YC (1994). Detection of β-glucosidase activity in polyacrylamide gels with esculin as substrate. Appl Environ Microbiol.

[CR23] Lynd LR, Weimer PJ, Zyl WHV, Pretorius IS (2002). Microbial cellulose utilization: Fundamentals and biotechnology. Microbiol Mol Biol Rev.

[CR24] Meier-Kolthoff JP, Auch AF, Klenk HP, Göker M (2013). Genome sequence-based species delimitation with confidence intervals and improved distance functions. BMC Bioinform.

[CR25] Meleiro LP, Salgado JCS, Maldonado RF, Alponti JS, Zimbardi ALRL, Jorge JA, Ward RJ, Furriel RPM (2015). A Neurospora crassa ß-glucosidase with potential for lignocellulose hydrolysis shows strong glucose tolerance and stimulation by glucose and xylose. J Mol Catal B Enzym.

[CR26] Murphy L, Bohlin C, Baumann MJ, Olsen SN, Sørensen TH, Anderson L, Borch K, Westh P (2013). Product inhibition of five *Hypocrea jecorina* cellulases. Enzyme Microb Technol.

[CR27] Nakazono-Nagaoka E, Fujikawa T, Shikata A, Tachaapaikoon C, Waeonukul R, Pason P, Ratanakhanokchai K, Kosugi A (2019). Draft genome sequence data of *Clostridium thermocellum* PAL5 possessing high cellulose-degradation ability. Data Brief.

[CR28] Nataf Y, Yaron S, Stahl F, Lamed R, Bayer EA, Scheper T-H, Sonenshein AL, Shoham Y (2009). Cellodextrin and laminaribiose ABC transporters in *Clostridium thermocellum*. J Bacteriol.

[CR29] Osborne CA, Galic M, Sangwan P, Janssen PH (2005). PCR-generated artefact from 16S rRNA gene-specific primers. FEMS Microbiol Lett.

[CR30] Patel BKC, Monk C, Littleworth H, Morgan HW, Daniel RM (1987) *Clostridium fervidus* sp. nov., a new chemoorganotrophic acetogenic thermophile. Int J Syst Evol Microbiol 37(2):123–126 10.1099/00207713-37-2-123

[CR31] Pei J, Pang Q, Zhao L, Fan S, Shi H (2012). *Thermoanaerobacterium thermosaccharolyticum* β-glucosidase: a glucose-tolerant enzyme with high specific activity for cellobiose. Biotechnol Biofuels.

[CR32] Plugge CM, Zoetendal EG, Stams AJ (2000). Caloramator coolhaasii sp. nov., a glutamate-degrading, moderately thermophilic anaerobe. Int J Syst Evol Microbiol.

[CR33] Prawitwong P, Waeonukul R, Tachaapaikoon C, Pason P, Ratanakhanokchai K, Deng L, Sermsathanaswadi J, Septiningrum K, Mori Y, Kosugi A (2013). Direct glucose production from lignocellulose using *Clostridium thermocellum* cultures supplemented with a thermostable β-glucosidase. Biotechnol Biofuels.

[CR34] Puchart V, Šuchová K, Biely P (2021). Xylanases of glycoside hydrolase family 30 - An overview. Biotechnol Adv.

[CR35] Qi K, Chen C, Yan F, Feng Y, Bayer EA, Kosugi A, Cui Q, Liu YJ (2021). Coordinated β-glucosidase activity with the cellulosome is effective for enhanced lignocellulose saccharification. Bioresour Technol.

[CR36] Richter M, Rosselló-Móra R (2009). Shifting the genomic gold standard for the prokaryotic species definition. Proc Natl Acad Sci U S A.

[CR37] Salgado JCS, Meleiro LP, Carli S, Ward RJ (2018). Glucose tolerant and glucose stimulated β-glucosidases - A review. Bioresour Technol.

[CR38] Shikata A, Sermsathanaswadi J, Thianheng P, Baramee S, Tachaapaikoon C, Waeonukul R, Pason P, Ratanakhanokchai K, Kosugi A (2018). Characterization of an anaerobic, thermophilic, alkaliphilic, high lignocellulosic biomass-degrading bacterial community, ISHI-3, isolated from biocompost. Enzyme Microb Technol.

[CR39] Singh G, Verma AK, Kumar V (2016) Catalytic properties, functional attributes and industrial applications of β-glucosidases. 3 Biotech 6(1):3 10.1007/s13205-015-0328-z 10.1007/s13205-015-0328-z PMC4697909 28330074

[CR40] Singhania RR, Patel AK, Sukumaran RK, Larroche C, Pandey A (2013). Role and significance of beta-glucosidases in the hydrolysis of cellulose for bioethanol production. Bioresour Technol.

[CR41] Sørensen A, Lübeck M, Lübeck PS, Ahring BK (2013). Fungal Beta-glucosidases: a bottleneck in industrial use of lignocellulosic materials. Biomolecules.

[CR42] Srivastava N, Rathour R, Jha S, Pandey K, Srivastava M, Thakur VK, Sengar RS, Gupta VK, Mazumder PB, Khan AF, Mishra PK (2019). Microbial beta glucosidase enzymes: Recent advances in biomass conversation for biofuels application. Biomolecules.

[CR43] Strobel HJ, Caldwell FC, Dawson KA (1995). Carbohydrate transport by the anaerobic thermophile Clostridium thermocellum LQRI. Appl Environ Microbiol.

[CR44] Tamura K, Dudley J, Nei M, Kumar S (2007) MEGA4: molecular evolutionary genetics analysis (MEGA) software version 4.0. Mol Biol Evol 24(8):1596–1599 10.1093/molbev/msm092 10.1093/molbev/msm092 17488738

[CR45] Teugjas H, Väljamäe P (2013). Selecting β-glucosidases to support cellulases in cellulose saccharification. Biotechnol Biofuels.

[CR46] Thompson JD, Higgins DG, Gibson TJ (1994). CLUSTAL W: improving the sensitivity of progressive multiple sequence alignment through sequence weighting, position-specific gap penalties and weight matrix choice. Nucleic Acids Res.

[CR47] Waeonukul R, Kosugi A, Prawitwong P, Deng L, Tachaapaikoon C, Pason P, Ratanakhanokchai K, Saito M, Mori Y (2013). Novel cellulase recycling method using a combination of *Clostridium thermocellum* cellulosomes and *Thermoanaerobacter brockii* β-glucosidase. Bioresour Technol.

[CR48] Waeonukul R, Kosugi A, Tachaapaikoon C, Pason P, Ratanakhanokchai K, Prawitwong P, Deng L, Saito M, Mori Y (2012). Efficient saccharification of ammonia soaked rice straw by combination of *Clostridium thermocellum* cellulosome and *Thermoanaerobacter brockii* β-glucosidase. Bioresour Technol.

[CR49] Yang F, Yang X, Li Z, Du C, Wang J, Li S (2015) Overexpression and characterization of a glucose-tolerant β-glucosidase from *T. aotearoense* with high specific activity for cellobiose. Appl Microbiol Biotechnol 99(21):8903–8915 10.1007/s00253-015-6619-9 10.1007/s00253-015-6619-9 25957152

[CR50] Yi Z-L, Zhang S-B, Pei X-Q, Wu Z-L (2013). Design of mutants for enhanced thermostability of β-glycosidase BglY from *Thermus thermophilus*. Bioresour Technol.

[CR51] Yoon SH, Ha SM, Lim J, Kwon S, Chun J (2017). A large-scale evaluation of algorithms to calculate average nucleotide identity. Anton Van Leeuwenhoek.

[CR52] Zhang J, Liu S, Li R, Hong W, Xiao Y, Feng Y, Cui Q, Liu YJ (2017). Efficient whole-cell-catalyzing cellulose saccharification using engineered *Clostridium thermocellum*. Biotechnol Biofuels.

[CR53] Zhang Y-HP, Lynd LR (2005). Cellulose utilization by *Clostridium thermocellum*: bioenergetics and hydrolysis product assimilation. Proc Natl Acad Sci U S A.

[CR54] Zhao L, Pang Q, Xie J, Pei J, Wang F, Fan S (2013). Enzymatic properties of *Thermoanaerobacterium thermosaccharolyticum* β-glucosidase fused to Clostridium cellulovorans cellulose binding domain and its application in hydrolysis of microcrystalline cellulose. BMC Biotechnol.

