# Supplementary material for: Biological cellulose saccharification using a coculture of Clostridium thermocellum and Thermobrachium celere strain A9
Source: Appl Microbiol Biotechnol. 2022 Feb 14;106(5-6):2133–45. doi: 10.1007/s00253-022-11818-0 (PMC8930880; doi:10.1007/s00253-022-11818-0)
Supplement: Supplementary file 1 — ESM1 (PDF 129 KB) [file 253_2022_11818_MOESM1_ESM.pdf]

Applied Microbiology and Biotechnology

Biological Cellulose Saccharification using a Coculture of *Clostridium thermocellum* and *Thermobrachium celere* strain A9 that Secretes Extracellular β-Glucosidase

Sreyneang Nhim[a], Rattiya Waeonukul[a,b], Ayaka Uke[c], Sirilak Baramee[a,b], Khanok Ratanakhanokchai[a], Chakrit Tachaapaikoon[a,b], Patthra Pason[a,b], Ya-Jun Liu[d,e,f] and Akihiko Kosugi[c]*

[a] Enzyme Technology Laboratory, School of Bioresources and Technology, King Mongkut's University of Technology Thonburi (KMUTT), Bangkok, 10150, Thailand

[b] Excellent Center of Enzyme Technology and Microbial Utilization, Pilot Plant Development and Training Institute (PDTI), King Mongkut's University of Technology Thonburi (KMUTT), Bangkok, 10150, Thailand

[c] Biological Resources and Post-harvest Division, Japan International Research Center for Agricultural Sciences (JIRCAS), 1-1 Ohwashi, Tsukuba, Ibaraki 305-8686, Japan

[d] CAS Key Laboratory of Biofuels, Qingdao Institute of Bioenergy and Bioprocess Technology, Chinese Academy of Sciences, Qingdao 266101, PR China

[e] Shandong Energy Institute, Qingdao 266101, PR China

[f] Qingdao New Energy Shandong Laboratory, Qingdao 266101, PR China

Corresponding author: Akihiko Kosugi
akosugi@affrc.go.jp
Tel & fax: +81-29-838-6625

Table S1. Comparison of physiological properties of strain A9 and similar bacteria.

Property	*Thermobrachium celere* strain A9	*Thermobrachium celere* strain JW/YL-NZ35	*Caloramator indicus* strain IndiB4[T]	*Caloramator fervidus* strain Rt4-B1[T]
Culture collection	NITE P-03545	DSM 8682	ACM 3982	ATCC 43204
Cell width/length (μm)	0.2–0.8/5–10	0.5–0.8/1.5–13	0.6–0.8/10–100	0.65–0.75/2.0–3.0
Gram straining	Positive	Positive	Negative	Negative
Spore formation	Negative	Negative	Negative	Positive
Optimum growth temp (°C)	60	66	60–65	68
Optimum growth pH	7.0–7.2	8.2	7.5–8.1	7.0–7.5
DNA G+C content (mol%)	31	30–31	25.6	39
Fermentation products	H_2, CO_2, acetate, ethanol	CO_2, H_2, acetate, formate, ethanol	Ethanol, acetate, lactate, CO_2, H_2	Acetate, CO_2, H_2, valerate, butyrate, ethanol, lactate
Substrate use				
Glucose	+	+	+	+
Cellobiose	+	−	+	N.D
Xylose	+	N.D*	N.D	+
Arabinose	+	N.D	N.D	−
Sucrose	+	+	+	−
Lactose	+	N.D	+	N.D
Starch	+	N.D	+	+
Xylan	+	N.D	N.D	+

Reference	This study	Engle *et al*. 1996	Chrisostomos *et al*. 1996	Patel *et al*. 1987

*N.D; not detected.
